# Case Report: Relapsing systemic lupus erythematosus treated with dual rituximab and anifrolumab therapy

**DOI:** 10.3389/fmed.2025.1727404

**Published:** 2026-01-06

**Authors:** Sunnie Lee, Janet Choi, Steven Benitez, Jeanie Lee

**Affiliations:** 1 Department of Medicine, Rutgers New Jersey Medical School, Newark, NJ, United States; 2 Division of Dermatology, Department of Medicine, Albert Einstein College of Medicine, Bronx, NY, United States; 3 Department of Radiology, Albert Einstein College of Medicine, Bronx, NY, United States; 4 Division of Rheumatology, Department of Medicine, Albert Einstein College of Medicine, Bronx, NY, United States

**Keywords:** anifrolumab, biologic therapies, case report, cutaneous lupus erythematosus, rituximab, Rowell syndrome, systemic lupus erythematosus, moyamoya disease

## Abstract

While biologic monotherapy is commonly used in the management of moderate-to-severe systemic lupus erythematosus (SLE), limited data exists regarding the efficacy and safety of dual biologic therapy in SLE. We report the case of a 42-year-old woman with a history of SLE complicated by Rowell Syndrome (RS) that was managed on anifrolumab and a recent diagnosis of moyamoya disease, who presented with new-onset lower extremity weakness. Laboratory testing was notable for active lupus serologies, and magnetic resonance angiography (MRA) findings were consistent with worsening moyamoya syndrome. Given the clinical and radiographic findings, a diagnosis of CNS lupus was suspected. Anifrolumab was discontinued and she was started on pulse dose corticosteroids and rituximab, resulting in marked neurologic improvement. However, she subsequently experienced a flare of RS. Anifrolumab was reintroduced while she remained on rituximab, leading to significant clinical improvement of her cutaneous symptoms. To our knowledge, we present the first case of successful treatment of refractory SLE with both cutaneous and neurologic involvement utilizing dual biologic therapy with rituximab and anifrolumab.

## Introduction

Biologic monotherapy is often used in the management of moderate-to-severe systemic lupus erythematosus (SLE), particularly in patients who exhibit an inadequate response to standard immunosuppressive agents. However, little is known about the efficacy and safety of dual biologic therapy in SLE. We present the first reported case of combined rituximab and anifrolumab therapy for refractory SLE in a patient with cutaneous and neuropsychiatric manifestations.

## Case presentation

A 42-year-old woman with a longstanding history of SLE presented to the hospital with acute onset of left leg weakness for 4 days. She was diagnosed with SLE in 2012 during a hospitalization for pericardial effusion and was treated with corticosteroids, hydroxychloroquine, and azathioprine. Her disease remained in remission for 8 years, during which azathioprine was tapered off while hydroxychloroquine monotherapy was continued. However, she subsequently developed lupus flares presenting with serositis and discoid lupus that failed to be controlled on belimumab.

Nine months prior to the current presentation, she developed Rowell Syndrome (RS) as a complication of SLE. She was diagnosed with RS based on the criteria proposed by Zeitouni et al., which includes presence of three major criteria—SLE, characteristic erythema multiforme-like lesions, and positive anti-nuclear antibody with a speckled pattern, along with one minor criteria of positive anti-Ro antibodies (1). She was started on mycophenolate mofetil but discontinued due to transaminitis. Anifrolumab was initiated and resulted in significant improvement of her cutaneous disease. However, she continued to experience recurring episodes of serositis and uveitis upon steroid taper.

Four months after the diagnosis of RS, she was hospitalized for slurred speech and right-hand weakness, found to have a multifocal left middle cerebral artery infarct. Laboratory studies at that time revealed elevated anti-double-stranded DNA (dsDNA) antibodies (74 U/ml) and decreased complement levels (C3 of 47 mg/dl and C4 of 6 mg/dl). Magnetic resonance angiography (MRA) of the head showed severe stenosis of left greater than right supraclinoid internal carotid arteries consistent with moyamoya disease (Supplementary Figures 1A-C). CNS lupus was considered but the presence of collaterals indicated progressive and slow terminal internal carotid artery vessel narrowing, which were more typical for non-inflammatory moyamoya disease than CNS vasculitis. In addition, vasculitis in SLE typically involves arterioles and capillaries rather than larger proximal vessels. Antiphospholipid syndrome was ruled out because she had negative lupus anticoagulant, anti-cardiolipin IgG and IgM, and beta-2 glycoprotein IgG and IgM antibodies. She subsequently underwent encephaloduroarteriosynangiosis and recovered without complications. Azathioprine was added while she remained on anifrolumab with goals to treat her serositis and uveitis. She had no other significant past medical history and no family history of autoimmune or neurological disease. She denied alcohol or tobacco use.

**Figure 1:**
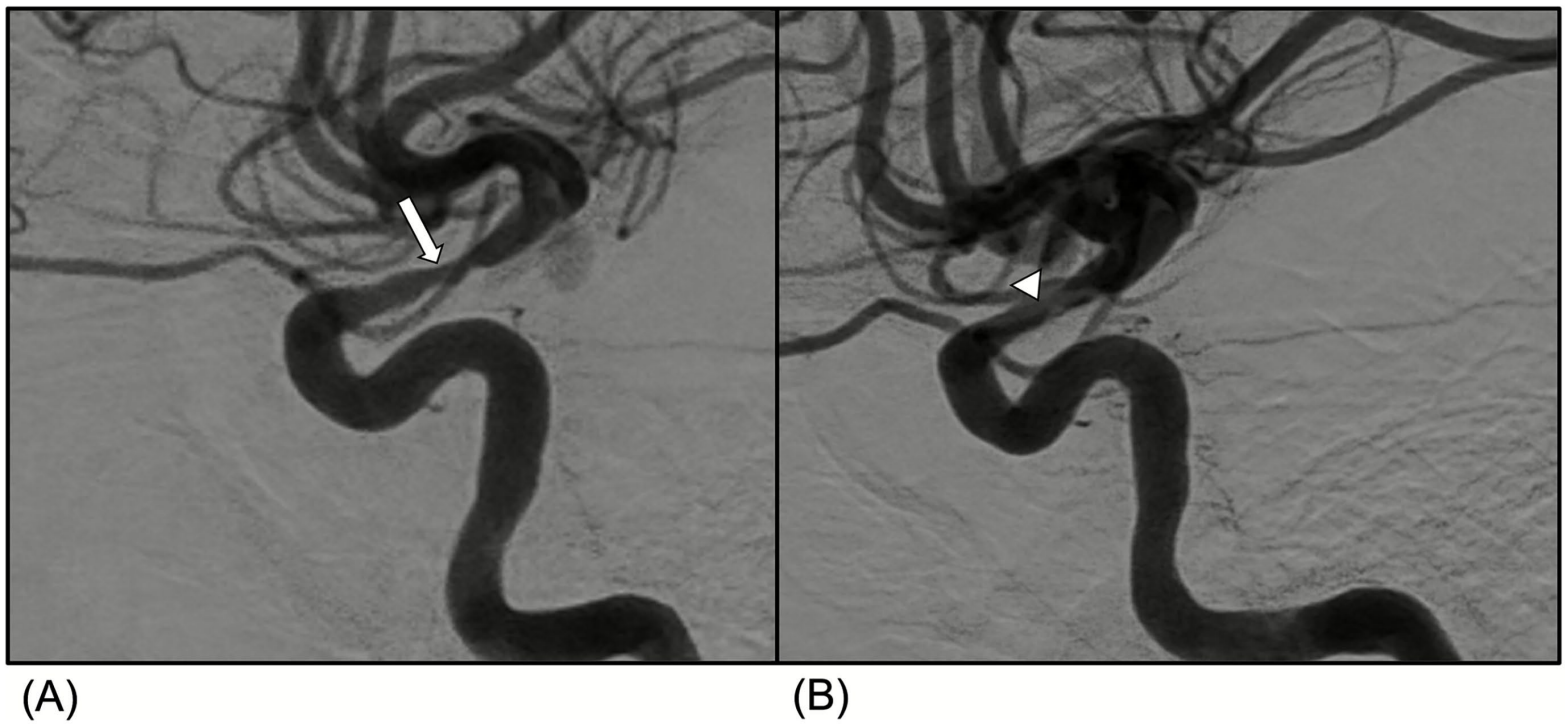
Radiologic findings of moyamoya syndrome. **(A)** Digital Subtraction Angiography (DSA) demonstrates progressive worsened narrowing of the right supraclinoid ICA (arrow) as compared with **(B)** a DSA study performed approximately 5 months earlier (arrowhead).

**Figure 2:**
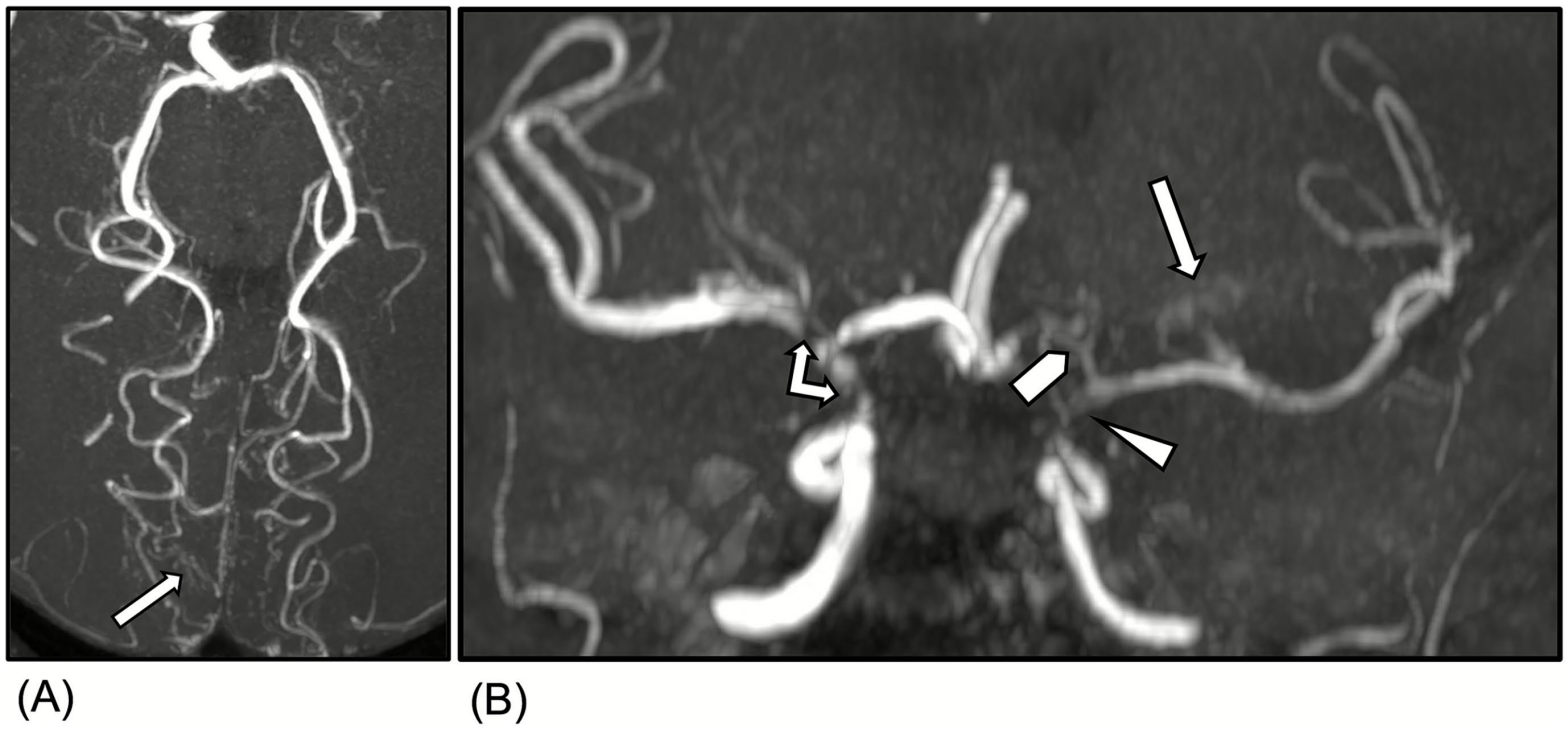
Magnetic Resonance Angiography of the head showed increased collaterals arising from the posterior cerebral arteries (**A**, arrow) and lenticulostriate vessels (**B**, arrow), severe stenosis/near occlusion of the left supraclinoid internal carotid artery (ICA) (**B**, arrowhead), severe stenosis of the right supraclinoid ICA and ICA terminus (**B**, double-headed arrow), stenosis/near occlusion of the left A1 segment (**B**, block arrowhead). Overall findings consistent with moyamoya syndrome.

On current presentation, which was 5 months since the patient’s admission for stroke, her neurological examination demonstrated left leg weakness with muscle strength of 4/5. She also had mild right faciobrachial paresis attributed to her prior left middle cerebral artery infarct. No rashes or mucosal lesions were observed. Laboratory data showed positive anti-nuclear antibody (>1:1280 speckled), elevated anti-dsDNA of 63 U/ml, and low complement levels (C3 of 44 mg/dl and C4 of 6 mg/dl). She also had positive anti-Smith antibody (>309 U/ml), RNP antibody (>217 U/ml), and SSA antibody (>239 U/ml). Inflammatory markers were normal with c-reactive protein of 0.5 mg/dl and erythrocyte sedimentation rate of 14 mm/h. Digital subtraction angiography (DSA) demonstrated worsened narrowing of the right supraclinoid internal carotid artery (ICA) when compared to studies from 5 months earlier (Figures 1A,B). MRA revealed increased collaterals arising from the posterior cerebral arteries and lenticulostriate vessels and severe stenosis of the left supraclinoid ICA and left A1 segment, which were consistent with moyamoya syndrome (Figures 2A,B).

Her recurrent strokes and progression of ICA stenosis and collaterals in the setting of persistently active lupus serologies raised concern for CNS lupus manifesting as a secondary moyamoya syndrome. She was initiated on pulse intravenous (IV) methylprednisolone 1 g daily for 3 days. Anifrolumab was discontinued due to inadequate CNS treatment of SLE and switched to rituximab 1 g IV followed by a second dose 2 weeks later, and then every 6 months. Hydroxychloroquine 400 mg daily was continued and she was transitioned to oral methylprednisolone at 1 mg/kg daily on discharge. She experienced marked neurological improvement following therapy.

**Figure 3:**
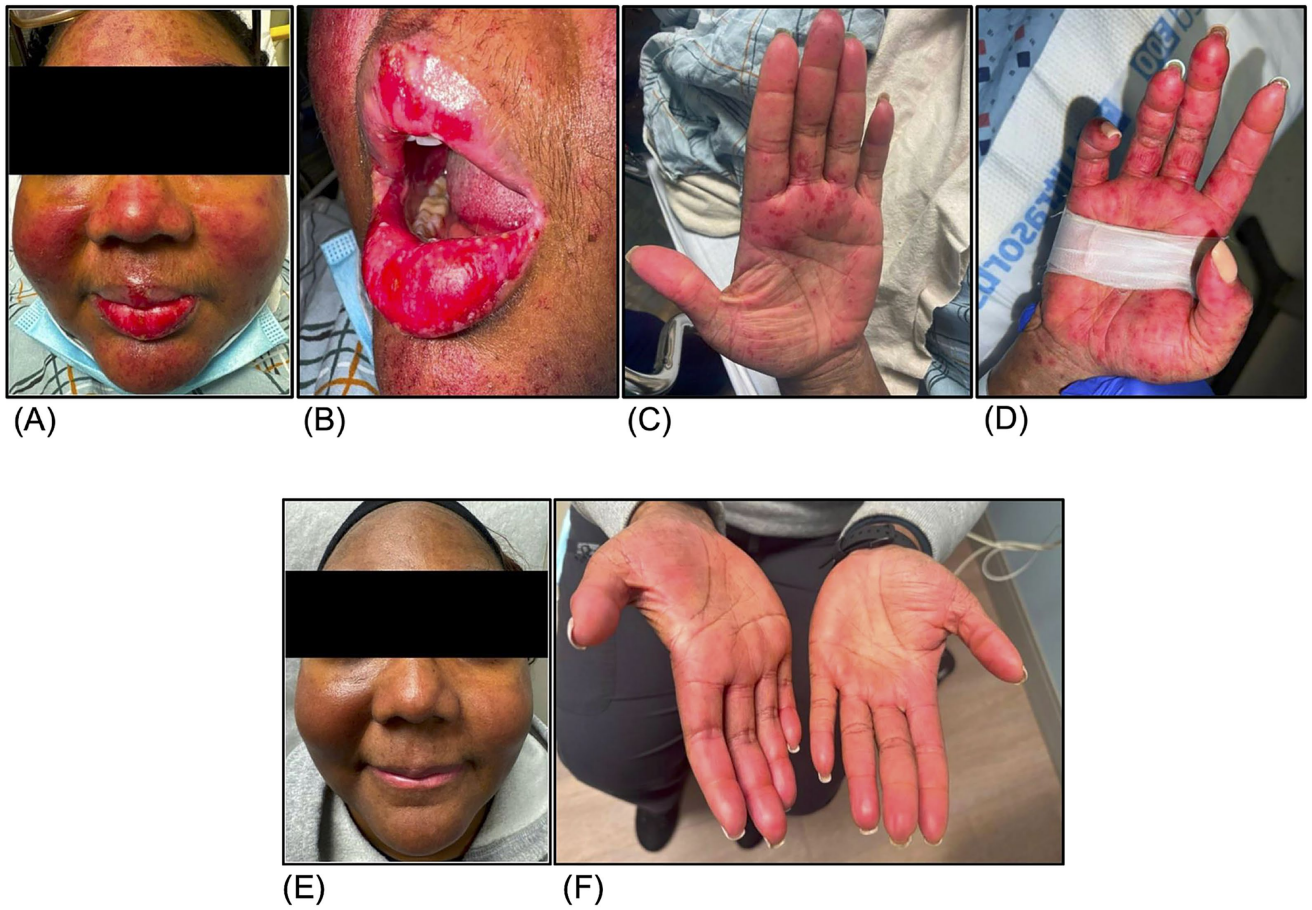
Rowell Syndrome lesions characterized by red to violaceous targetoid macules and papules on the face **(A)**, lips **(B)**, left palm **(C)**, and right palm **(D)** before dual rituximab and anifrolumab therapy. Resolution of Rowell Syndrome lesions as shown on face **(E)** and palmar surfaces of the hands **(F)** 5 months after dual rituximab and anifrolumab therapy.

Two months after the discontinuation of anifrolumab, however, the patient was readmitted with a flare of her RS characterized by painful red-to-violaceous targetoid macules and papules in a malar distribution of her face, scalp, chest, arms, hands, as well as erosive oral and vulvar lesions (Figures 3A–D). Anifrolumab 300 mg monthly was reinitiated while she remained on rituximab 1 g every 6 months. She continued the dual biologic therapy after discharge and was able to successfully taper off her steroids.

**Figure 4:**
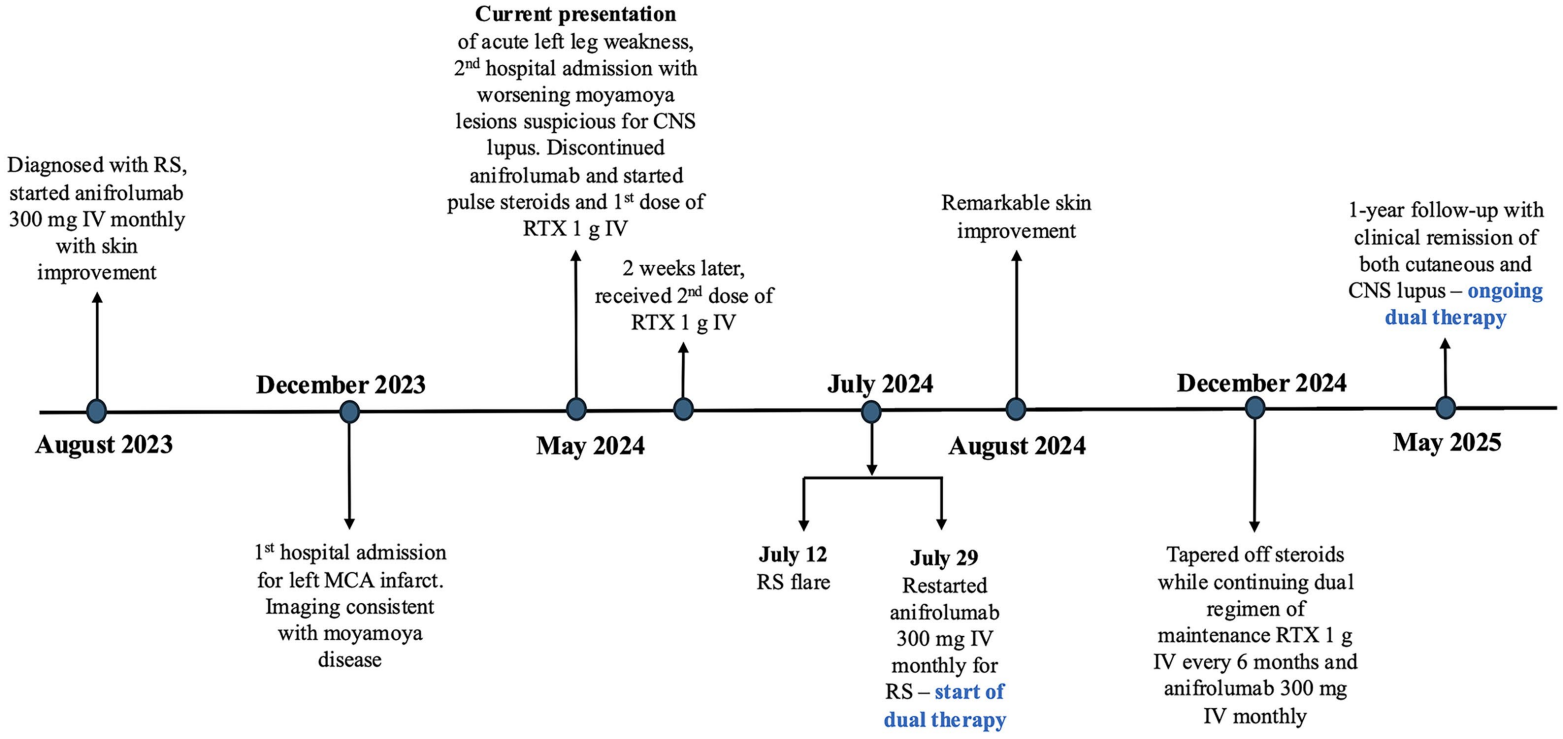
Key events timeline. RS, Rowell syndrome, MCA, middle cerebral assay, CNS, central nervous system, RTX, rituximab.

At one-year follow-up, she remained in clinical remission with resolution of both her CNS and cutaneous symptoms (Figures 3E,F) and normalization of her serologies with negative anti-dsDNA (2 U/ml) and normal complement levels (C3 of 109 mg/dl and C4 of 31 mg/dl). A follow-up computed tomography angiography of the head showed stable moyamoya lesions. Through participation in physical therapy, she regained strength in her left leg and recovered her ability to ambulate independently. There were no infections or adverse events on this regimen. The timeline of key clinical events, including therapy initiation, discontinuation, and outcomes is summarized in Figure 4.

## Discussion

SLE is a chronic autoimmune disease that can have multiple organ involvement, including the skin and CNS. RS is a rare variant of cutaneous lupus erythematosus (CLE) associated with SLE, characterized by erythema multiforme-like lesions and distinctive serologic criteria. A few case reports, including the present case, have demonstrated that anifrolumab can effectively treat RS that is refractory to conventional therapies (2–4).

Anifrolumab is a human monoclonal antibody that binds to subunit 1 of the type 1 interferon receptor (IFNAR1) and inhibits type 1 interferon (IFN-1) signaling. The IFN-1 pathway plays a central role in the pathogenesis of SLE and CLE. Patients with CLE, with or without concurrent SLE, often exhibit increased expression of type 1 IFN-regulated genes in their peripheral blood, which correlates with cutaneous disease activity (5).

Despite remarkable improvement of her cutaneous symptoms on anifrolumab, our patient developed a neuropsychiatric lupus flare presenting as secondary moyamoya syndrome. Moyamoya syndrome is a rare cerebrovascular complication of SLE, described in a limited number of case reports. This condition is thought to result from CNS vasculitis leading to progressive large cerebral vessel occlusions and subsequent formation of collateral vessels (6, 7). While corticosteroids remain the first-line therapy of CNS lupus, rituximab, a chimeric monoclonal antibody targeting CD20-positive B-cells, is utilized in refractory cases (8).

This case highlights the potential limitations of biologic monotherapy in the treatment of complex SLE manifestations caused by impairments in multiple pathways. Specifically, anifrolumab alone may be insufficient for treating CNS lupus, while rituximab alone may not adequately control cutaneous lupus. The patient achieved disease control only after receiving combined therapy of rituximab and anifrolumab.

The concurrent use of anifrolumab and rituximab presents theoretical risks of additive immunosuppression, which may increase susceptibility to serious bacterial and opportunistic infections and hypogammaglobulinemia. Anifrolumab targets IFN-1, which regulates the development of plasma cells, myeloid dendritic cells, and upregulation of T-cells that are important for defense against viruses primarily through the innate immune system. On the other hand, rituximab binds to CD20 on B cells and induce B-cell depletion through the adaptive immune system (9). Since the two drugs inhibit different pathways, dual therapy may be administered safely but requires close monitoring for infections. Our patient received zoster, pneumococcal conjugate and influenza vaccines, and did not develop any infections on the combination therapy.

Current evidence regarding the combined use of biologic therapies in SLE is limited. Some studies suggest the combination of rituximab and belimumab may be effective in treating refractory SLE cases (10). To our knowledge, there are no prior reports of combined rituximab and anifrolumab therapy. Hence, this is the first case demonstrating the efficacy and safety of dual therapy with rituximab and anifrolumab in refractory SLE.

### Patient perspective

The patient expressed tremendous satisfaction with the dual therapy and reported that “my lupus flares are nonexistent nowadays for a full year.” She was most concerned about the painful oral ulcers and rash on her face during her RS flares, as well as the steroids that “made me bloated from face down.” On this regimen, she has been able to taper off steroids and stated that her confidence and mood have improved since achieving disease remission. She also reported she did not face any challenges during the treatment and would highly recommend the combination therapy to other patients.

## Data Availability

The original contributions presented in the study are included in the article/Supplementary material, further inquiries can be directed to the corresponding author.
