# Supplementary material for: Case Report: Relapsing systemic lupus erythematosus treated with dual rituximab and anifrolumab therapy
Source: Front Med (Lausanne). 2026 Jan 6;12:1727404. doi: 10.3389/fmed.2025.1727404 (PMC12816201; doi:10.3389/fmed.2025.1727404)
Supplement: Supplementary file 1 [file Image_1.pdf]

Supplementary Figure

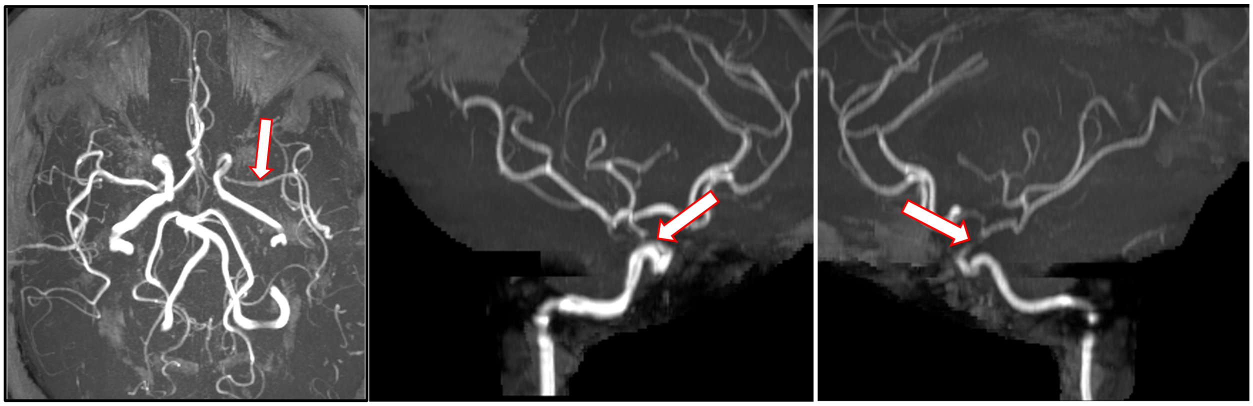
(A) (B) (C)

Supplementary Figure 1. Magnetic Resonance Angiography of the head demonstrates attenuated flow-related signal along the left middle cerebral artery (**A**) and marked narrowing of the bilateral supraclinoid internal carotid arteries (**B, C**)
